# High-throughput 3D microvessel-on-a-chip model to study defective angiogenesis in systemic sclerosis

**DOI:** 10.1038/s41598-022-21468-x

**Published:** 2022-10-08

**Authors:** Bart Kramer, Claudio Corallo, Angelique van den Heuvel, Justin Crawford, Thomas Olivier, Edo Elstak, Nicola Giordano, Paul Vulto, Henriette L. Lanz, Richard A. J. Janssen, Michela A. Tessari

**Affiliations:** 1 Mimetas BV, Leiden, The Netherlands; 2 Galapagos BV, Leiden, The Netherlands; 3 Galapagos Biopharma Italy S.R.L, Milano, Italy; 4 Scleroderma Unit, Department of Medicine, Surgery and Neurosciences, University of Siena, Siena, Italy; 5 Present Address: Genmab BV, Utrecht, The Netherlands

**Keywords:** Peripheral vascular disease, Assay systems, Tissue engineering

## Abstract

In early systemic sclerosis (Scleroderma, SSc), the vasculature is impaired. Although the exact etiology of endothelial cell damage in SSc remains unclear, it is hypothesized that endothelial to mesenchymal transition (EndoMT) plays a key role. To perform physiologically relevant angiogenic studies, we set out to develop an angiogenesis-on-a-chip platform that is suitable for assessing disease parameters that are relevant to SSc and other vasculopathies. In the model, we substituted Fetal Bovine Serum (FBS) with Human Serum without impairing the stability of the culture. We showed that 3D microvessels and angiogenic factor-induced sprouts exposed to key pro-inflammatory and pro-fibrotic cytokines (TNFα and TGFβ) undergo structural alterations consisting of destructive vasculopathy (loss of small vessels). We also showed that these detrimental effects can be prevented by compound-mediated inhibition of TGFβ-ALK5 signaling or addition of a TNFα neutralizing antibody to the 3D cultures. This demonstrates that our in vitro model is suitable for compound testing and identification of new drugs that can protect from microvascular destabilization or regression in disease-mimicking conditions. To support this, we demonstrated that sera obtained from SSc patients can exert an anti-angiogenic effect on the 3D vessel model, opening the doors to screening for potential SSc drugs, enabling direct patient translatability and personalization of drug treatment.

## Introduction

Systemic Sclerosis (Scleroderma, SSc) is a chronic autoimmune connective tissue disease characterized by vasculopathy, inflammation and progressive fibrosis of the skin and internal organs, such as lungs, heart, kidneys and gastrointestinal tract^1^. Vascular alteration is an early and central event in SSc pathogenesis, and it usually precedes the onset of fibrosis^2^. Endothelial cells, as central constituents of the vascular system, play a key role in all aspects of vascular homeostasis as well as in physiological or pathological processes like thrombosis, inflammation, and vascular wall remodeling^3^. Although the etiology of vascular damage in SSc remains unclear, different hypotheses have been formulated to explain this phenomenon^4^. Among these, autoimmunity and the so-called endothelial to mesenchymal transition (EndoMT) appear to play a key role^5,6^. In fact, anti-endothelial cell antibodies have been found in numerous autoimmune and/or inflammatory/infectious diseases. These conditions include, in addition to SSc, rheumatoid arthritis, systemic lupus erythematosus, polymyositis, several forms of vasculitis, and cytomegalovirus infection, among others^7,8^. In vitro investigations^9^ demonstrate that SSc-specific autoantibodies embedded in immune complexes induce a pro-inflammatory and pro-fibrotic phenotype at the endothelial level. Incubation of endothelial cells with SSc-immune complexes results in modulation of several molecules involved in the three cardinal scleroderma pathophysiological processes. Firstly, it can lead to vascular dysfunction, where a key vascular alteration is represented by the critical imbalance between factors promoting vasoconstriction (e.g., endothelin ET-1) and vasodilation (e.g., nitric oxide). Secondly, SSc-immune complexes can inflame the perivascular space, with aberrant cytokine and chemokine release, and overexpression of adhesion molecules, including intercellular adhesion molecule-1 (ICAM-1) and vascular cell adhesion molecule-1 (VCAM-1)^10^. Thirdly, microvascular fibroproliferative lesions and other abnormalities associated with altered secretion of growth factors and profibrotic cytokines, such as transforming growth factor-beta (TGFβ), can develop. TGFβ is a profibrotic cytokine that plays a key role in the ligand-mediated receptor process that triggers the onset and progression of SSc^11^.

Once activated, endothelial cells contribute to disease pathogenesis by mediating the fibroproliferative vasculopathy characteristic of SSc: the unbalanced production of vasoactive mediators resulting in vasoconstriction; the increased expression of adhesion molecules by damaged endothelial surfaces promoting leukocyte diapedesis, activation, and accumulation; endothelial cell transdifferentiation into myofibroblasts gaining mesenchymal cell markers (EndoMT)^12^.

Onset of pathophysiological processes in SSc results in impaired vascular homeostasis, because of which angiogenesis is dysregulated and efficient vascular recovery is impaired^13^. The result is the onset of avascular areas and tissue hypoxia that leads to complications such as digital ulcers^14^, pulmonary arterial hypertension^15^, and fibrosis^16^.

Most of the experimental systems for studying angiogenesis in SSc rely on animal models^17^. The first in vitro models were developed using two-dimensional cell culture-based assays^18^. Scientists demonstrated that human microvascular endothelial cells (HMVECs) cultured on a two-dimensional gelatinous protein mixture resembling the extracellular matrix (ECM) can form capillary-like structures upon treatment with pro-angiogenic factors^19^. They also demonstrated that disease relevant cytokines (e.g. ET-1, TGFß) or SSc sera can compromise capillary formation^20^, which could be partially restored using selective compounds^21,22^.

These two-dimensional in vitro models allow the study of fundamental endothelial cell biology, such as migration, proliferation and capillary formation^23^. However, two-dimensional models also have several limitations, including their limited throughput (capillary morphogenesis is usually performed in 6- or 24-well plates) and the short-term stability of the capillary-like structures (24 to 48 h). This only allows for the study of tubulogenesis, but not the stability of the formed tubules. Additionally, these assays do not recapitulate the angiogenesis process including its typical hallmarks, such as perfused lumen formation, differentiation into tip and stalk cells, anastomosis and widening of lumen^24^. Progress in tissue engineering and the advent of microfluidic-assisted tissue engineering has resulted in more sophisticated three-dimensional (3D) in vitro models for angiogenesis studies^25^. Although these models better recapitulate the angiogenesis conditions in vivo, they are quite complex to establish. In addition, they are usually implemented on single chips, thus compromising proper experimental design with multiple conditions (e.g. replicates, dilutions, controls)^26^. Only few setups are compatible with routine testing and screening requirements, such as high content imaging (HCI)-based multiplexed cellular and molecular analyses, automated liquid handling and real time measurements^27^.

Recently, a gradient-driven, three-dimensional angiogenesis assay in a standardized microfluidic platform was described using immortalized human umbilical vein endothelial cells (HUVECs)^28^ or induced pluripotent stem cells (iPS cells)^29^. In this assay, angiogenic sprouting is induced from a perfused main vessel through a patterned collagen-1 gel. The resulting angiogenic sprouts have clear lumen, tip-stalk cell hierarchy and undergo anastomosis and vessel widening upon prolonged culture. In this work, we utilize this platform to study angiogenesis in primary human microvascular endothelial cells (HMVECs) and assess disease parameters that are relevant to SSc and other vasculopathies. We show that 3D microvessels and angiogenic factor-induced sprouts exposed to key pro-inflammatory and pro-fibrotic cytokines (TNFα and TGFβ) undergo structural alterations typical of destructive vasculopathy (loss of small vessels). We investigate how compound-mediated inhibition of TGFβ-ALK5 signaling or addition of TNFα neutralizing antibodies can prevent these detrimental effects. In addition, we demonstrate that sera obtained from SSc patients can exert an anti-angiogenic effect on the 3D vessel model, thus opening the doors for direct patient translation and personalization of the assay. Our in vitro model is suited for compound testing and identification of new drugs that can protect from microvascular destabilization and regression in disease-mimicking conditions.

## Material and methods

### Cell culture

Primary HMVEC (Human Microvascular Endothelial Cells) (Lonza CC-2543) were cultured in regular T75 culture flasks (Corning, 734–2705) with EBM2 medium (Lonza CC-3156) containing the EGM-2MV kit (Lonza-4147). HMVEC were passaged one time before being seeded into the OrganoPlate® 3-lane (MIMETAS 4003–400-B, Leiden, The Netherlands). Cell detachment was done with Trypsin/EDTA solution 0.25 mg/mL (Lonza CC-5012) and neutralized with Trypsin Neutralization solution TNS (Lonza CC-5002).

### OrganoPlate culture

We used the OrganoPlate 3-lane with 320 µm (top and bottom perfusion channels) × 360 µm (middle gel channel) × 220 µm (w × h) channels (MIMETAS 4003–400-B, Leiden, The Netherlands). Gel and perfusion channel lengths are 2.2 mm and the Phaseguides have dimensions of 100 µm × 55 µm (w × h). Before gel seeding, 50 µL of Hank’s balanced salt solution (HBSS) was dispensed into the observation window to prevent evaporation and enhance optical clarity. A stock solution of 5 mg/mL rat tail collagen type I (Cultrex rat collagen 1, 5 mg/mL; Trevigen, Gaithersburg, MD, USA) was neutralized with 10% 37 g/L NaHCO3 (Sigma, S5761) and 10% 1 M HEPES buffer (Gibco, 15,630–056) to obtain a concentration of 4 mg/mL. The neutralized collagen was kept on ice until use and used within 10 min. Using a repeater pipette, 2 µL of the neutralized collagen was added into the inlet of each gel channel. To polymerize the collagen, the OrganoPlate was incubated for 10 min at 37 °C, 5% CO2. After incubation, the device was removed from the incubator and 30 µL HBSS was added to the gel inlet. The OrganoPlate was stored in the incubator (37 °C, 5% CO_2_) until cell loading next day.

HMVEC were trypsinized with Trypsin/EDTA solution 0.25 mg/mL and neutralized with Trypsin Neutralization solution TNS. Cells were pelleted and resuspended at a concentration of 10^6^ cells/mL in EBM2 medium containing EGM-2MV kit. HBSS in the gel inlet was aspirated right before cell loading. 2 µL of the cell suspension was dispensed into the top perfusion inlet and incubated for 5 h at 37 °C, 5% CO_2_ in the OrganoPlate plate stand to allow the cells to attach to the ECM. After the cells attached to ECM, 50 µL of medium was added in the top perfusion inlet and outlet wells. The plates were placed in the incubator (37 °C, 5% CO_2_) on a rocking platform with a 4-min interval at an angle of 7°. Next day a medium change was performed by replacing the EBM2 medium with EGM-2MV kit for EBM2 medium with adjusted EGM-2MV kit where the FBS in the kit was replaced with 2% (final concentration) Human Healthy Serum (HHS, Tebu-bio, HSER-10ML). The serum consists of 3 donors pooled (1 male, 2 female, average age 35 ± 13.1 years).

### Sprout formation, stimulation with angiogenic factors

HMVEC vessels were cultured for 3 days, in medium with HHS, before a gradient of angiogenic factors was applied. The bottom perfusion channels of the OrganoPlate 3-lane were washed with EBM2 medium (30 µL of medium per well) for 10 min. Stock solutions of the angiogenic factors were prepared as follows: 100 µg/mL hVEGF-165 (Preprotech, 100–20) in 0.1% BSA in PBS, 1 mM Sphingosine-1-Phosphate (S1P, Sigma, G00918) in 5% 1 M HCl, 95% DMSO, and 10 µg/mL Phorbol Myristate Acetate (PMA, Sigma, P1585) in 0.1% DMSO/MilliQ. Angiogenic factors were diluted in EBM2/EGM-2MV/HHS culture medium and used at the following concentrations: 50 ng/mL hVEGF-165, 2 ng/mL PMA, and 500 nM S1P. All medium was aspirated and fresh medium without angiogenic cocktail was added to the top perfusion channel (15 µL per in/outlet) while medium with the angiogenic cocktail was added to the bottom perfusion channel (15 µL per in/outlet). Angiogenic sprouts were monitored by phase contrast microscopy until ready for assays which was after 4 days of stimulation.

After 4 days of sprout stimulation, the medium with angiogenic cocktail was replaced with basal medium (Lonza CC-3156) without angiogenic cocktail containing antibiotics (GA-1000 from EGM-2MV kit), HHS at 2% or 5% final concentration.

### Sprout stability, exposure, and rescue

#### Stability

For the stability experiments, after 4 days of sprout formation the angiogenic cocktail medium was replaced by imaging medium (basal medium with antibiotics, HHS or HPS and Calcein-AM) for 30 min to establish the baseline and imaged (see sprout visualization and quantification section). After imaging, the imaging medium was replaced with basal medium containing antibiotics (GA-1000) and 2% HHS, 5% HHS or 5% HPS from several donors. After 48 h, the sprouts were incubated for 30 min with basal medium containing Calcein-AM (1:2000, 15 µL per top or bottom in/outlet) before imaging and assessing the sprout stability without angiogenic cocktail present in the medium.

#### Exposure and rescue

For exposure experiments, the medium with angiogenic cocktail was replaced with basal medium without angiogenic cocktail containing antibiotics, HHS at 2% or 5% final concentration, Calcein-AM (1:2000 diluted) and inhibitory compounds for exposure: Alk5i 10 mM final concentration (Sigma, S4317) and TNFα neutralizing antibody 0.32 µg/mL final concentration (TNFα iAb, Invivogen Cat# Htnfa-mab1). Medium was applied in top and bottom in- and outlets (15 µL per in/outlet). After 30 min incubation, the cultures were imaged and used as timepoint zero for further analysis. After 90 min, the pre-incubation medium was exchanged for exposure medium. Exposure medium contained: basal medium, GA-1000 antibiotics, HHS (5% final concentration), HPS (2% or 5% final concentration) and exposure compounds or proper vehicle controls in different combinations and concentrations. For the triggers, TGFβ II at 10 ng/mL final concentration (R&D systems, Cat#: 302-B2) and TNFα at 10 ng/mL final concentration (ImmunoTools, Cat#: 11,343,015) were used as well as a combination of the two at equal concentrations.

#### Patients’ enrollment

Serum samples were collected from 8 female patients (age, 56.9 ± 13.4 years; disease duration, 5.9 ± 4.5 years) affected by diffuse-cutaneous-SSc (dSSc) (n = 4), by limited-cutaneous SSc (lSSc) (n = 3) and by SSc-sine-scleroderma (ssSSc) (n = 1) diagnosed in accordance with LeRoy et al. 1988 and who fulfilled the 2013 American College of Rheumatology/European League Against Rheumatism diagnostic criteria for SSc^30^. All patients gave their fully informed, voluntary, written consent according to the principles of the Declaration of Helsinki and in compliance with the ethics committee of the University of Siena, whose institutional review board approved the entire study protocol with code GLPG SSc 16. The major demographic and clinical characteristics of the patients enrolled are shown in Table 1.

**Table 1:** The demographic and clinical characteristics of the patients enrolled in this study.

Patient	Age (years) Sex	Disease duration (years)	Digital ulcers	PAH	Capillaroscopic pattern	ANA-ENA	Drug treatment
71 dSSc	52 F	10	Yes	No	Active	Antinucleolar-Scl-70	lloprost, Bosentan, glucocorticoids
72 ISSc	34 F	1.5	No	No	Active	Antinucleolar-Scl-70	Glucocorticoids
74 ISSc	42 F	0.5	No	No	Active	Antinucleolar-Scl-70	Nifedipine
75 dSSc	61 F	10	No	No	Active	Antinucleolar-Scl-70	lloprost, glucocorticoids, MMF
77 ssSSc	70 F	10	Yes	Yes	Active	Antinucleolar-Scl-70	lloprost, Bosentan, Nifedipine
79 dSSc	71 F	10	Yes	Yes	Active-late	Antinucleolar-Scl-70	Macitentan, MMF, Nifedipine
SO dSSc	67 F	1	No	Yes	Active	Antinucleolar-Scl-70	Macitentan, MMF, glucocorticoids
83 ISSc	58 F	4	No	No	Active	Antinucleolar-Scl-70	Glucocorticoids, Nifedipine, Azathioprine
Mean (SD)	56.9 (13.4)	5.9 (4.5)					

MMF, Mycophenolate mofetil; SD, Standard deviation.

### Immunohistochemistry

#### Fixation

HMVEC tubes and sprouts were fixated using 3.7% formaldehyde (Sigma, 252,549) in HBSS (Sigma H6648) for 15 min, washed twice with HBSS for 5 min and stored with 50ul HBSS per well at room temperature until immunofluorescent staining.

#### Staining

HMVEC tubes were washed with washing solution containing 4% Fetal Calf Serum (FCS) (Gibco, cat# A13450) in PBS (Gibco, cat# 70,013,065) for 5 min and permeabilized with 0.3% Triton X-100 (Sigma, T8787) in PBS for 10 min. After permeabilization, the cells were washed with washing solution for 5 min before the blocking buffer, containing 2% FCS, 2% BSA (Sigma, cat# A2153), and 0.1% Tween20 (Sigma, cat# P9616) in PBS, was added for 50 min. After blocking, the cells were immediately incubated for 2 h with a primary antibody in blocking solution, washed two times for 3 min with washing solution and incubated with a secondary antibody in blocking solution, including a nuclear stain, for 30 min. All steps were performed at room temperature. Primary antibodies used were Rabbit anti-human VE-Cadherin 1:1000 (Abcam, Ab33168), Mouse anti-human αSMA 1:1000 (Sigma, A2547), Mouse anti-human CD31 1:20 (Dako, M0823) and Rabbit anti-human SM22 (Abcam, Ab14106). The secondary antibodies were donkey-anti-mouse Alexa Fluor 647 (Invitrogen, A31571) and goat-anti-rabbit Alexa Fluor Plus 488 (Invitrogen, A32731). For nuclear staining Hoechst 33,342 (Thermo Fisher Scientific H3570) 1:2000 was used.

### Sprout visualization and quantification

All images were captured with an ImageXPress XLS-C HCI system (Molecular Devices) & ImageXPress IXM-XLS micro HCI (Molecular Devices). During cell culture, phase-contrast (4X, 0.13NA) images were obtained on the IXM-XLS to monitor growth of the HMVEC tubules over time. These images were processed in Fiji^31^ to enhance contrast to aid with visual interpretation of the status of the cultures.

To visualize and quantify the growth of the endothelial sprouts, a fluorescent dye (Calcein-AM – FITC excitation & emission) was added to the HMVEC tubules at specific timepoints. These tubules were then imaged on the IXM-C HCI microscope. To accurately capture the 3D-nature of the tubules a 10X 0.5NA, FITC excitation / emission & a Nipkow 60 µm spinning disk was used to capture Z-stacks of the tubules. The separate Z-slices were stored to allow for 3D-reconstruction of the Z-data. Maximum intensity projections (MIP) were also created to quantify the growth of the tubules.

The MIP images were loaded into Fiji and subjected to a rolling ball background correction^32^. The resulting image was subjected to a threshold using Li’s Minimum Cross Entropy threshold^33^ to extract the area of the image covered by endothelial sprouts. The ratio of area of staining before & after compound addition was then calculated. This metric was then averaged over all replicates of a given condition and compared against the mean of other conditions. Statistics were performed with the ‘one-way ANOVA’ function in GraphPad Prism version 6.

## Results

### Endothelial microvessel culture and sprouting in a microfluidic platform

**Figure 1:**
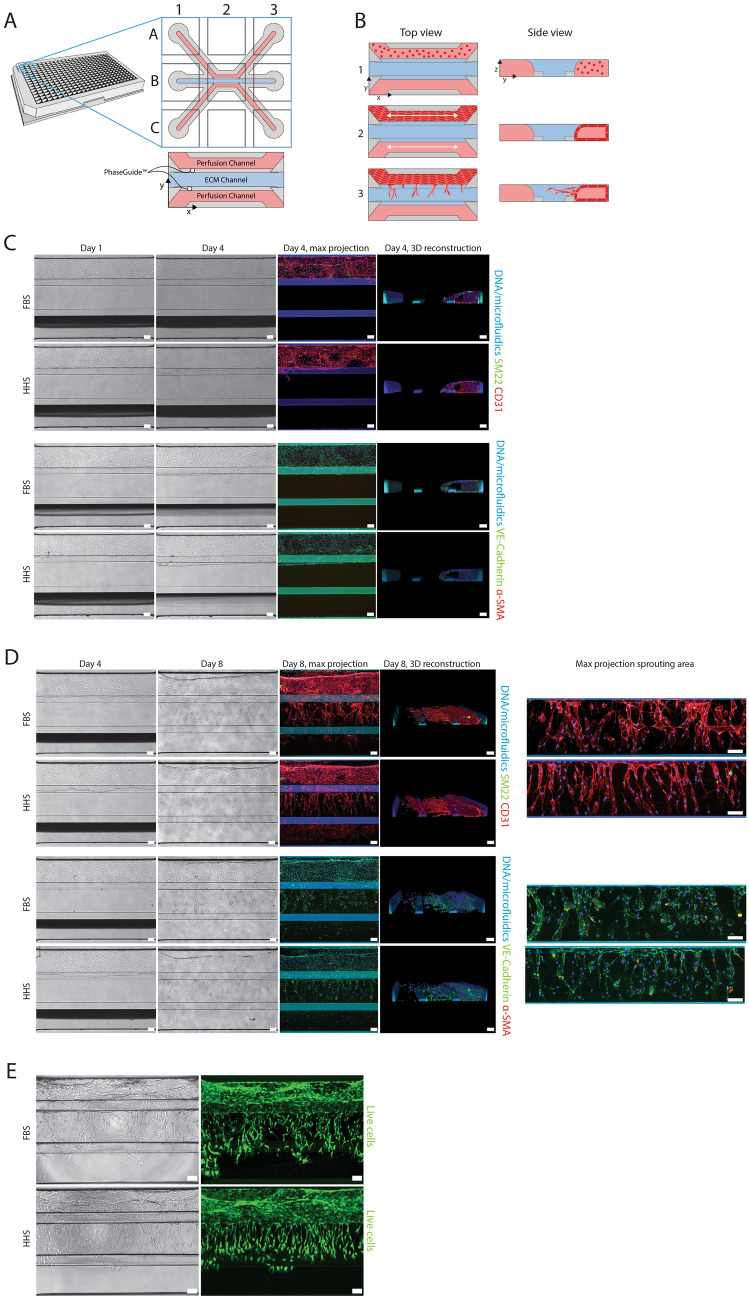
Endothelial microvessel culture and sprouting. (**A**) The microfluidic microtiter plate ‘OrganoPlate’ was used for a 3D cell culture, based on a 384 well plate interface with 40 microfluidic chips integrated in the bottom. The gel channel (blue) holds the collagen extracellular matrix (ECM) in place through the phaseguide’s pressure barrier function. (**B**) Endothelial cells are loaded in the top channel (perfusion lane) to form a microvessel adjacent to the ECM in the middle channel. Bidirectional perfusion of the culture is induced by placing the OrganoPlate on an interval rocking platform. A gradient of an angiogenic cocktail is applied to induce sprout formation. (**C**) Immunofluorescent characterization of HMVEC tubules after 4 days of culture with FBS or HHS. (**D**) Immunofluorescent characterization of HMVEC tubules after 4 days of sprouting with FBS or HHS. On the right, a confocal maximum projection of the middle channel represents the sprouting area in the ECM. (**E**) Calcein-AM live cell staining of sprouted HMVEC cultures on day 8. All scale bars are 100 µm.

HMVECs were cultured in the 3-Lane OrganoPlate® (Fig. 1A). The process of vessel formation and subsequent angiogenic sprouting is depicted in Fig. 1B. First, HMVECs were seeded in the top channel of the chip (1) and allowed to form a three-dimensional tubular structure (2). Bidirectional flow was introduced into the system by placing the plate on a rocking device. To induce angiogenic sprouts, a cocktail of pro-angiogenic factors was introduced into the bottom channel (3).

HMVECs were cultured as tubules for 4 days. The medium was supplemented with FBS or HHS (Fig. 1C). The substitution of FBS for HHS prepares the model for the subsequent exposure to patient serum. To verify endothelial phenotype, HMVECs were stained with various markers. Tubules were positive for two endothelial-junction-associated protein markers, vascular endothelial (VE)-Cadherin and CD-31 (PECAM-1), and negative for the myofibroblast markers α-smooth muscle actin (α-SMA) and SM22α by visual assessment. A 3D reconstruction revealed that in both serum conditions, the cells formed a fully confluent tubule without invasion in the collagen-I ECM (not stained).

After the microvessels reached confluency on day 4, an angiogenic sprouting cocktail containing the growth factors hVEGF-165, S1P and PMA, was applied to the bottom perfusion channel. Due to the perfusion of the culture by placement of the culture vessel on a rocking platform, a stable gradient of angiogenic factors was formed which stimulated the HMVEC tubules to develop sprouts towards the bottom perfusion channel ^28^. Figure 1D depicts the culture after 4 days of sprouting and supplementary Fig. 1 shows the whole sprouting process over time. Angiogenic sprouts including tip and stalk cells were observed spanning the full ECM channel directing towards the source of the gradient. A 3D reconstruction reveals that sprouting occurs on different z-levels. No obvious differences in the expression patterns of CD31, VE-Cadherin, SM22 and α-SMA were observed between cultures treated with FBS or HSS, indicating that in the established model, human serum can be used without affecting the expression of endothelial cell-specific markers. Next, live cultures were stained with the cell-permeant dye Calcein AM. In live cells, the non-fluorescent dye is converted to a green fluorescent calcein, which visualizes all live cells in the culture (see Fig. 1E). This allows the observation and quantification of the angiogenic sprouts over time.

### Quantitative tracking of sprout initiation

**Figure 2:**
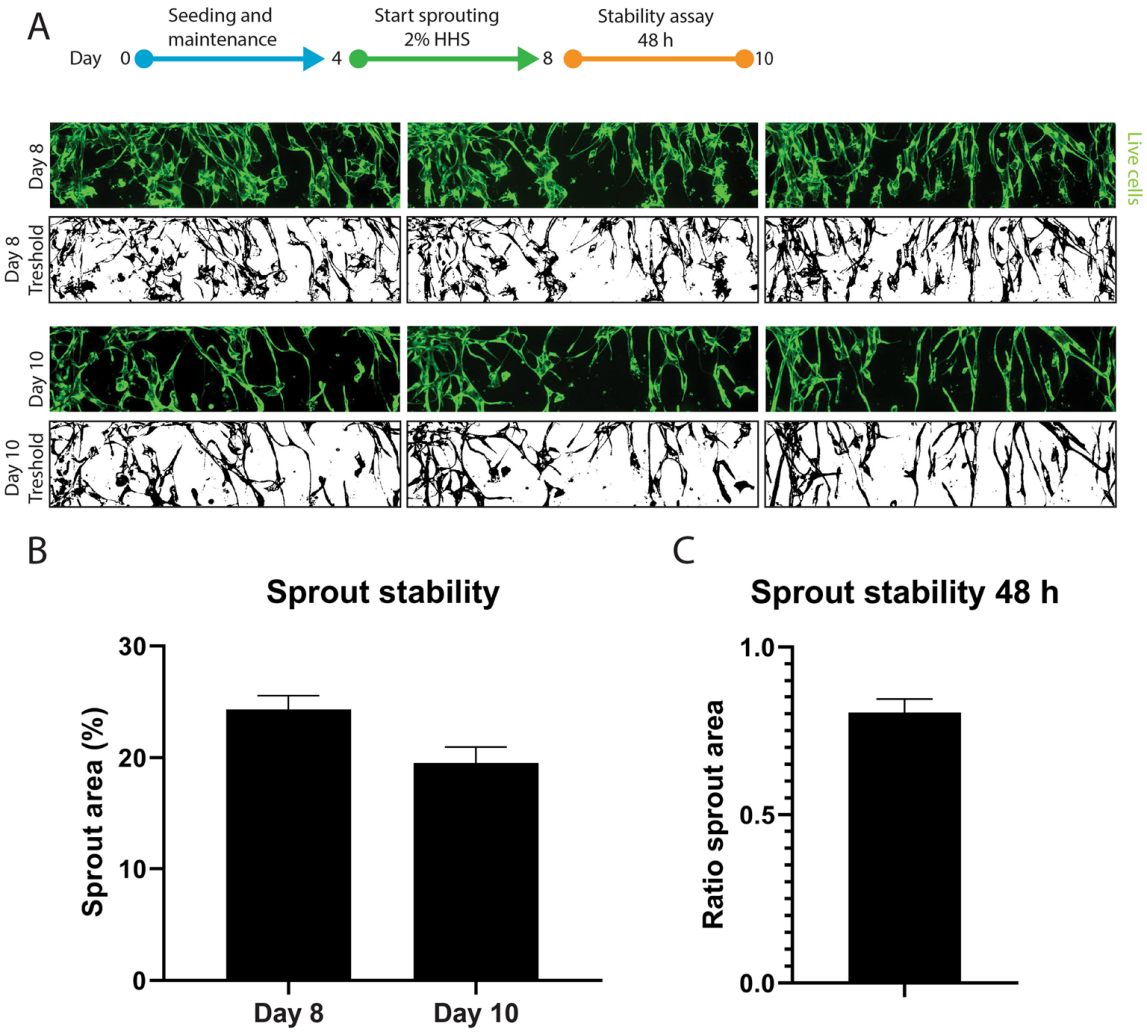
Stability assessment of sprouted HMVEC microvessels. (**A**) Representative maximum projection confocal images (top) and threshold images (bottom) used for quantification of the sprouting area. (**B**) Quantification of the area covered by sprouts at the start (day 8) and end (day 10) of the stability assay (n = 9, represented is mean ± SEM). (**C**) Ratio of the area covered by sprouts at day 10 versus day 8 (n = 9, shown is mean ± SEM).

To quantitively follow the angiogenesis process over time, we measured the coverage of calcein stained sprouts in the ECM channel (Fig. 2A). To assess the stability of the culture for compound exposure, the angiogenic cocktail was withdrawn from day 8 till day 10 (48 h) and the sprout area was measured. A small decrease in sprout area was observed (24% area at day 8, 20% area at day 10, Fig. 2B, ratio in Fig. 2C) related to thinning of vessels, but no regression of sprouts was observed. This provided a 48-h window that can be utilized for compound or serum exposure.

### Response of sprouted microvessels to cytokines and inhibitors

**Figure 3:**
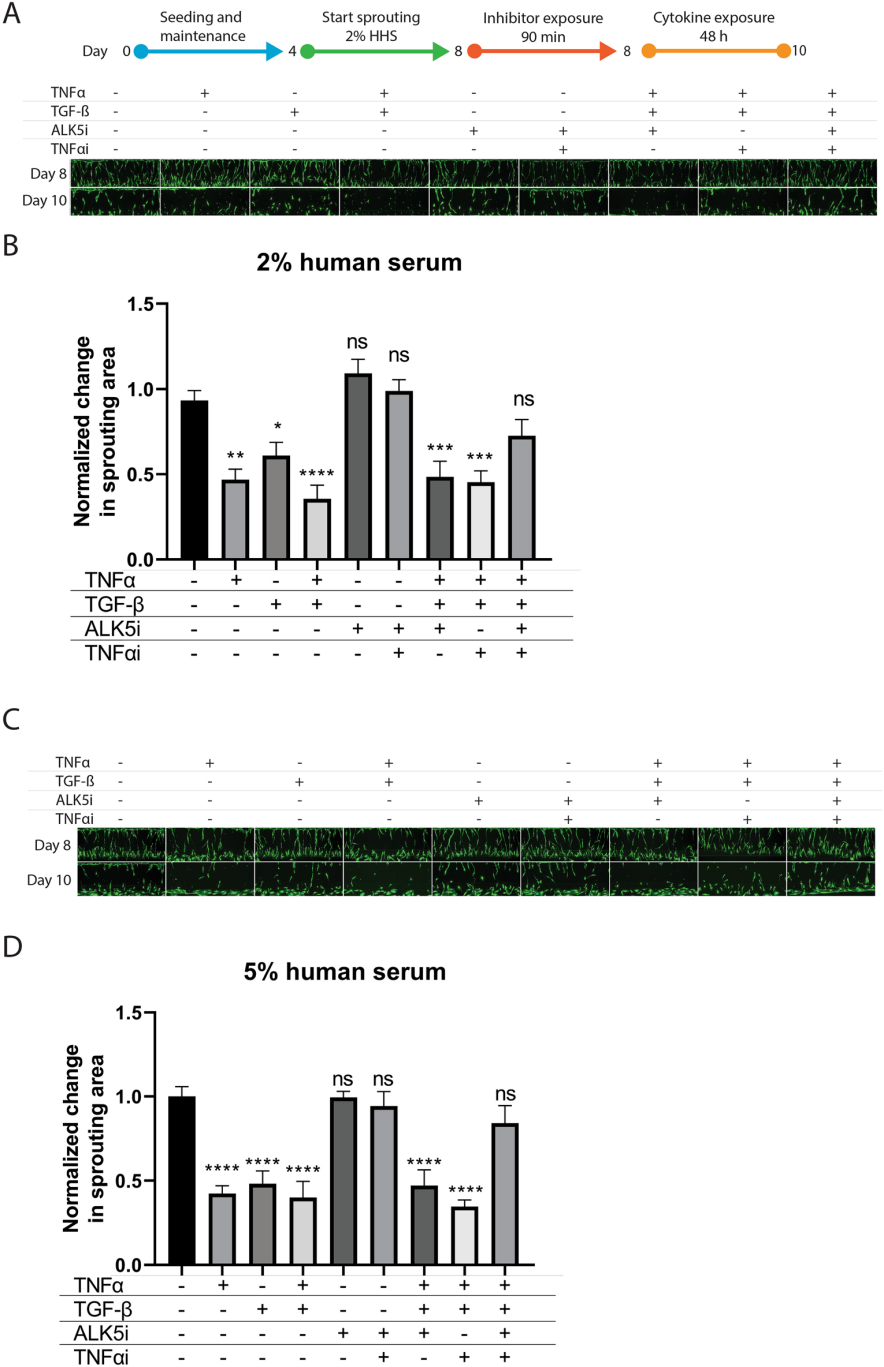
Response of the sprouted microvessels to cytokines and inhibitors. Sprouted microvessels were preincubated on day 8 with ALK5i or TNFα iAB for 90 min and subsequently exposed to (a combination of) TNFα or TGFß for 48 h in 2% (panel **A** and **B**) or 5% (panel **C** and **D**) human serum. (**A**) and (**C**) show representative images of cultures exposed to (a combination of) inhibitors and cytokines. (**B**) and (**D**) show quantification plots of the area covered by sprouts of exposed microvessels after exposure. The ratio of the sprouting area before and after the stability assay was normalized to the no exposure condition. n = 7–12, shown is the mean ± SEM, statistical test – ordinary one-way ANOVA (ns = not significant, * p < 0.05, ** p < 0.01, *** p < 0.001, **** p < 0.0001).

To induce a diseased in vitro state relevant to SSc and other vasculopathies, we assessed the stability of the established sprouts upon exposure to key pro-inflammatory (TNFα) and pro-fibrotic (TGFß) cytokines. Cytokines and inhibitors were added in combination with a low (2%) and high (5%) amount of HHS (Fig. 3). TGFß has been shown to induce full EndoMT in cultured endothelial cells from different tissues^34,35^, whereas serum levels of TNFα are elevated in patients with SSc and favor the development of pulmonary fibrosis and pulmonary arterial hypertension. TNFα inhibitors reduce systemic inflammation, improving the endothelial function and decreasing the risk of pulmonary arterial hypertension progression^36^. A significant reduction in sprout area was measured when adding TNFα (47%), TGFß (42%) or a combination of both cytokines (60%), indicating the degradation of the angiogenic sprouts after additions of these triggers (Fig. 3). The effect was observed in both human serum conditions (2% and 5% HHS).

Next, we investigated whether the cytokine-induced destabilization of the vascular structures could be prevented by inhibiting both TNFα and TGFß signaling (Fig. 3). TNFα signaling was inhibited by adding a neutralizing monoclonal antibody against human TNFα. TGFß was inhibited with the small molecule SB-431542, which inhibits TGFß-mediated activation of SMAD proteins by the ALK5 receptor. In the presence of the combined TNFα and TGFß trigger, the addition of the TNFα neutralizing antibody or SB-431542 alone did not result in any protection of the microvessels. However, when the HMVECs were incubated with both TNFα and TGFß inhibitors before exposure to the trigger, the sprout area was preserved and was found to be comparable to the no trigger control. This effect was observed in presence of both 2% (Fig. 3A + B, 2.3-fold reduction) or 5% (Fig. 3C + D, fourfold reduction) healthy human serum in the culture medium, although the rescue effect was slightly larger in the 5% human serum condition. The inhibition and rescue of fresh sprouts of dermal endothelial cells with cytokines showed that the developed assay has an adequate window for exposure, which can be utilized for the routine assessment of compounds and patient samples on angiogenic sprout formation.

### Model application: systemic scleroderma serum assessment

**Figure 4:**
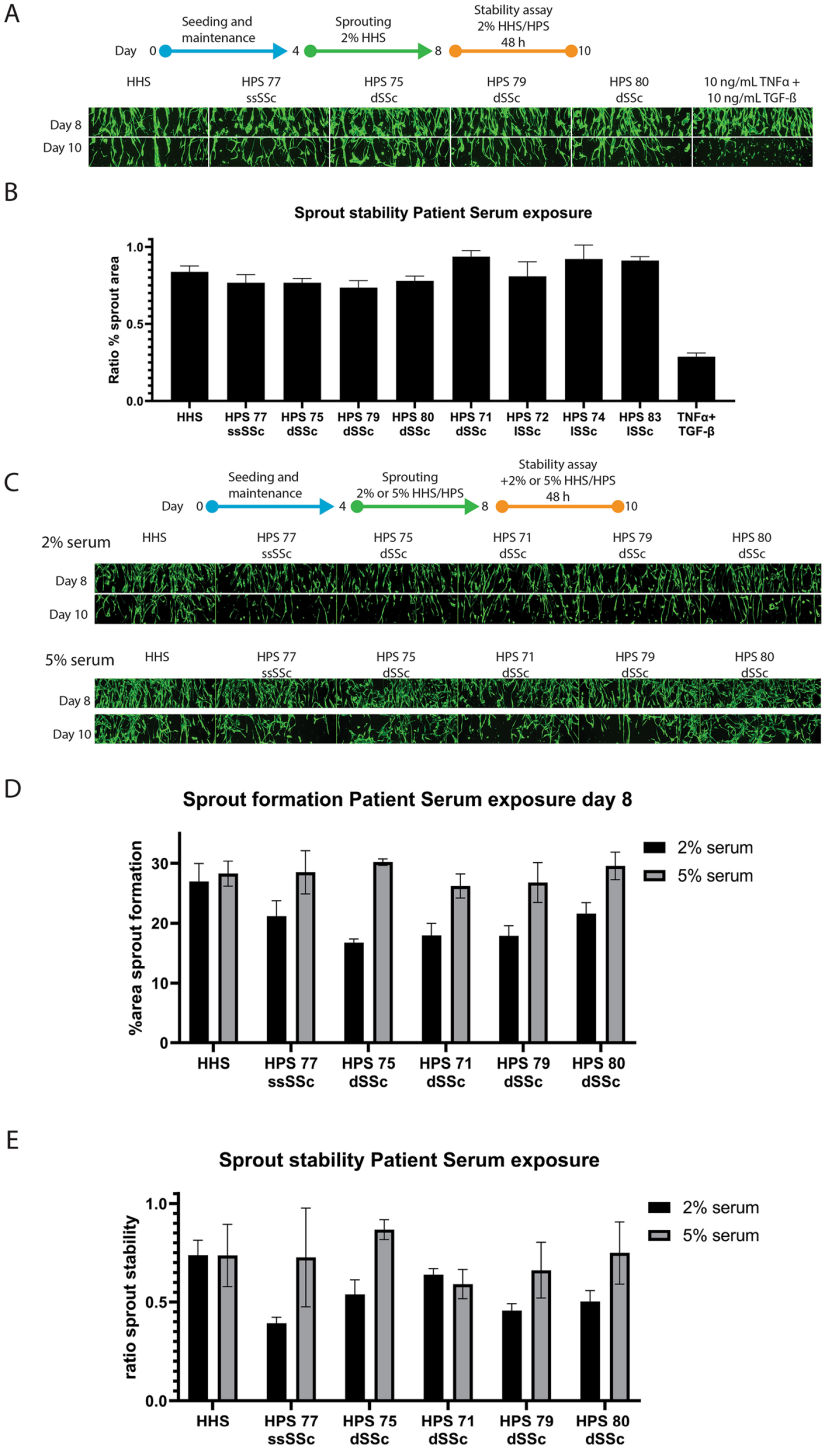
Exposure of sprouted HMVEC microvessels to sera from patients with systemic scleroderma. (**A**) Angiogenic sprouting was induced from day 4 till day 8 including 2% healthy human serum, followed by exposure for 48 h with 2% patient serum of 8 systemic scleroderma patients. Representative images before and after exposure are shown. (**B**) Bar graph presenting the ratio of the area covered by sprouts after versus before patient serum exposure. n = 3–6 shown is mean ± SEM. (**C-E**) Angiogenic sprouting was induced in the presence of 2% or 5% patient serum for 4 days. Subsequently, sprout stability was verified following four days of culture in absence of the sprouting cocktail with the addition of patient serum. Representative images are shown in (**C**), quantification of sprouting area at day 8 in (**D**) and the ratio of sprout stability in (**E**). n = 3–4, shown are mean ± SD.

Previous studies have demonstrated that treatment with sera from patients with SSc impairs the tubulogenic performance of human microvascular endothelial cells in an in vitro matrigel assay^37^. As an application of the developed angiogenesis-on-a-chip model, we assessed the effect of different human serum samples from scleroderma patients on the angiogenic sprout formation and stability. Serum samples from eight different SSc patients (Table 1) were interrogated. First, the sprouts were formed in HHS for 4 days. Subsequently, the patient serum was introduced at a 2% concentration to the microfluidic system during the 48-h stability assay (Fig. 4A + B). No significant reduction in sprout area could be observed after 48 h compared to the HHS control.

As the sprouts were formed from day 4 till day 8 in HHS (Fig. 4A + B) before exposure to patient serum, we hypothesized that an effect of the patient serum might be observed on sprout formation. We observed that sprouts formed in all exposure conditions. However, cultures exposed to 2% patient serum formed 31% less dense sprout networks at day 8 compared to the HHS control (Fig. 4C + D).

In addition, cultures exposed to 2% patient serum were on average 29% less stable than the healthy serum control after the 48-h stability assay (Fig. 4E). We observed that the cultures exposed with 2% patient serum were on average 28% less stable than cultures exposed with 5% patient serum.

## Discussion

There is a growing need for physiologically relevant assays to model complex diseases in an in vitro setting. Traditionally, animal models have been widely utilized to study disease, but animal models are expensive, labor-intensive, and suffer from ethical and biological limitations^38,39^. To alleviate the need for better models, scientists are increasingly embracing three-dimensional tissue culture to develop more physiologically relevant models^40^. Organ-on-a-chip technology offers an even higher degree of complexity, where cells are cued to form complex 3D structures in miniaturized channels^41^. The technology has been used to model a wide array of organs and processes, such as the blood–brain barrier^42^, kidney^43^, cancer^44,45^ and vasculature^46,47^ and is adopted widely by the pharmaceutical industry^48^. In this work, we developed and optimized a 3D microvessel-on-a-chip on the OrganoPlate platform to study angiogenesis in the context of systemic sclerosis (SSc). We believe this model could represent an optimal solution balancing decent throughput (40 independent samples on a single well plate compatible with automated liquid handlers) with a physiologically relevant model of angiogenesis, overcoming the limitations of 2D models characterized by non-physiologically relevant conditions of cultured cells, and of more complex 3D models restricted by lack of throughput.

We developed a quantitative readout, which allows assessment of both sprout formation and sprout stability. The assays were optimized for human serum culture, replacing the more traditionally used fetal bovine serum. We demonstrated that we could recapitulate typical hallmarks of SSc, such as microvascular destabilization and sprout regression through pro-fibrotic and pro-inflammatory cytokines TGFβ and TNFα, and showed that addition of inhibitors could prevent the diseased phenotype. Finally, we showed that we could study the effect of human sera derived from SSc patients on sprout formation and stability.

The usage of human serum as an alternative for bovine serum for endothelial cell culture has been proposed previously^49–51^, but not in the light of an angiogenesis assay. This study optimized the use of human serum in endothelial cell culture thereby allowing us to study the effects of human patient serum on the formation and stability of angiogenic sprouts without a confounding switch from bovine to human serum. In combination with a window for sprout stability of 24 h, this enabled a unique study on the effect of SSc patient derived serum on the stability of sprouts.

Microvascular destabilization or regression in SSc, leading to consequent dysregulated angiogenesis, could be induced by autoantibodies^9^ and by pro-fibrotic and pro-inflammatory cytokines such as TGFß and TNFα. However, it is not clear whether a particular cytokine, or the synergistic actions of groups of cytokines and/or autoantibodies could initiate the endothelial damage^9^. Hence, the need to develop an assay in which one or multiple triggers can be used to recapitulate the onset of the endothelial damage and relative inhibitors can be added to prevent or rescue the phenotype without losing the assay window, becomes fundamental. In our 3D microvessel-on-a-chip model, we managed to obtain an adequate assay window especially when TNFα alone or in combination with TGFß were used as triggers. We observed almost full prevention of 3D microvascular destabilization in presence of a TGFß receptor inhibitor and TNFα blocker, providing the opportunity to assess the effect of novel drugs.

As a proof of concept, we also assessed the effect of different serum samples from SSc patients and observed that patient sera predominantly influenced angiogenic sprout formation and stability when added during the formation of the sprouts. The assay window, however, was shorter compared to purified cytokines.

It needs mentioning though that all sera used in this study were collected from patients undergoing pharmacological treatments. This might have contributed to reduced effects in the angiogenesis assay. The discrepancy in assay window between sprout destabilization by purified cytokines (e.g., TNFα or TGFβ) and patients’ sera, might also suggest that these cytokines are present in lower concentrations in sera from patients with prolonged disease and treated with anti-inflammatory or anti-fibrotic drugs.

As a future perspective for further development of this assay, we foresee two improvements in the study design. The first one is the cell source: we used cells from healthy donors and not from SSc patients due to the hurdles of isolating them from skin biopsies. A model containing SSc cells could have been suitable for angiogenesis rescue experiments and would potentially increase the 48-h exposure window. The second improvement would be to include sera from SSc patients who were naïve to treatments, to potentially increase our assay window for sprouts formation and stability.

In conclusion, we described an angiogenesis-on-a-chip platform which, with 40 chips per microtiter plate, is suitable for high-throughput applications. We optimized the culture protocol to replace FBS with the physiologically more relevant HHS, to allow for assessment of patient sera. As a proof of concept, we investigated the effect of SSc patient serum on our platform and found a destabilizing effect on the angiogenic sprouts. Our in vitro assay allowed us to study and monitor all the stages of angiogenesis, from sprout formation to sprout stabilization and degradation in the presence of different triggers and compounds. We foresee further uptake of the assay in development of novel treatments against scleroderma.

## Supplementary Information

Supplementary Information 1.

Supplementary Information 2.

## Data Availability

The datasets generated during in this study are available from the corresponding authors on reasonable request.
