# Supplementary figures and images for: High-throughput 3D microvessel-on-a-chip model to study defective angiogenesis in systemic sclerosis
Source: Sci Rep. 2022 Oct 8;12:16930. doi: 10.1038/s41598-022-21468-x (PMC9547891; doi:10.1038/s41598-022-21468-x)

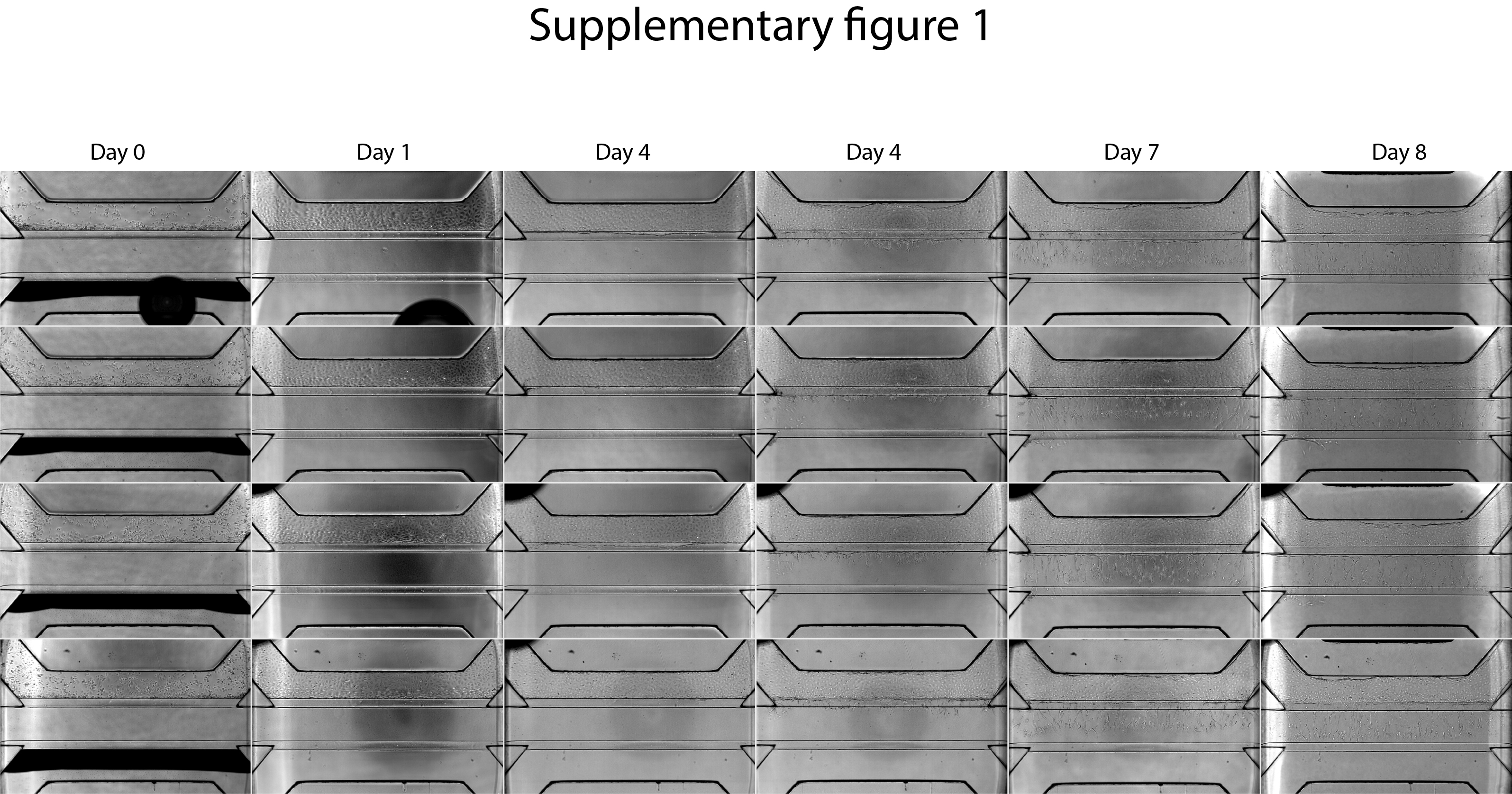

Supplement: Supplementary file 1 — Supplementary Information 1. [file 41598_2022_21468_MOESM1_ESM.jpg]
