# Supplementary material for: High-throughput 3D microvessel-on-a-chip model to study defective angiogenesis in systemic sclerosis
Source: Sci Rep. 2022 Oct 8;12:16930. doi: 10.1038/s41598-022-21468-x (PMC9547891; doi:10.1038/s41598-022-21468-x)
Supplement: Supplementary file 2 — Supplementary Information 2. [file 41598_2022_21468_MOESM2_ESM.docx]

**Supplementary figure 1** Angiogenic sprout formation over time in the OrganoPlate 3-lane. Cells were loaded into the top channel at day 0 and allowed to form a tubule. At day 4, sprouting was initiated. Shown are 4 representative cultures.
